# Overview of Radar Alignment Methods and Analysis of Radar Misalignment’s Impact on Active Safety and Autonomous Systems

**DOI:** 10.3390/s24154913

**Published:** 2024-07-29

**Authors:** Rafał Michał Burza

**Affiliations:** Department of Metrology and Electronics, AGH University of Kraków, al. A. Mickiewicza 30, B-1, 30-059 Kraków, Poland; burza@agh.edu.pl

**Keywords:** active safety, alignment, automotive, autonomous driving, calibration, misalignment, radar, vehicle

## Abstract

The rapid development of active safety systems in the automotive industry and research in autonomous driving requires reliable, high-precision sensors that provide rich information about the surrounding environment and the behaviour of other road users. In practice, there is always some non-zero mounting misalignment, i.e., angular inaccuracy in a sensor’s mounting on a vehicle. It is essential to accurately estimate and compensate for this misalignment further programmatically (in software). In the case of radars, imprecise mounting may result in incorrect/inaccurate target information, problems with the tracking algorithm, or a decrease in the power reflected from the target. Sensor misalignment should be mitigated in two ways: through the correction of an inaccurate alignment angle via the estimated value of the misalignment angle or alerting other components of the system of potential sensor degradation if the misalignment is beyond the operational range. This work analyses misalignment’s influences on radar sensors and other system components. In the mathematically proven example of a vertically misaligned radar, pedestrian detectability dropped to one-third of the maximum range. In addition, mathematically derived heading estimation errors demonstrate the impact on data association in data fusion. The simulation results presented show that the angle of misalignment exponentially increases the risk of false track splitting. Additionally, the paper presents a comprehensive review of radar alignment techniques, mostly found in the patent literature, and implements a baseline algorithm, along with suggested key performance indicators (KPIs) to facilitate comparisons for other researchers.

## 1. Introduction

Autonomous driving (AD), initially thought to be a matter of a decade of development, has proven to be the most challenging task for the automotive world. Technology constantly evolves to bring vehicle autonomy level 5 into the daily lives of the general population. To achieve that, microchip manufacturers are focused on developing more powerful computing units that include the acceleration of neural networks and the parallel processing (execution) of complex algorithms [1,2]. The demand for various functionalities in consumer vehicles accelerates the increase in system complexity in both the number of electronic control units (ECUs) and the size of software, bringing new challenges related to software engineering, cybersecurity and the complexity of the development process itself [3,4,5].

New radar designs are emerging to reduce the size and cost of devices. With such an aim, researchers developed an antenna-in-chip radar design for automotive applications [6]. Decreasing the cost of the production and size of a sensor while maintaining the required performance may make advanced driving assistance systems (ADASs) more feasible for the general public and enable the placement of an increased number of radars in a vehicle.

The increased coverage of the environment with cost-effective sensors increases the possible complexity of the system, but the autonomous revolution also requires careful consideration of how to design the road infrastructure so as not to hinder the capabilities of automotive sensors. Although sensors and actuators will usually provide faster reaction times than human drivers, a careful design of road curvature will increase the potential field of view (FoV) of a sensor, reducing the risk of braking with maximum vehicle capabilities and ultimately resulting in higher comfort for passengers [7].

Radars are also evolving with new antenna designs that, despite their relatively small size, can discriminate between both horizontal and vertical angles [8]. The increase in the computational power of microprocessors also makes it possible to process higher amounts of radar detections and transmit them to an ECU. This feature also requires a high-speed connection between the sensor and the ECU, which was recently made possible with the SOME/IP protocol using an Ethernet network, which achieves 100 Mbit/s with a possible 1 Gbit/s advance in the future [9].

While sensor designs are becoming more complex and increasing their capability, verifying a sensor’s state of health and reporting it to the system is still important. Sensor performance can be degraded due to an object that covers a sensor. In the case of corner sensors, an incorrectly positioned or painted bumper can hinder radar wave propagation or offset the wave. In those cases, vehicle manufacturers are responsible for accounting for such effects in vehicle design and placing sensors where fascia interference is minimal. Similar effects can also occur during the daily operation of a vehicle due to mud or snow covering the sensor, which requires an algorithmic approach to detecting sensor blockage [10].

AD and ADAS systems consisting of multiple sensors require sensor detections translated to a common vehicle coordinate system (VCS) with the origin of the vehicle chassis. This requires precise information about the sensor mounting position and mounting angle. Although, for some sensors, horizontal accuracy can achieve values greater than 1 degree—which satisfies the radar accuracy requirements for AD and ADASs [11]—mounting angle accuracy still poses a problem. Although vehicle manufacturers can take complex measures to increase mounting accuracy, it still requires validation and further algorithmic correction. The problem is magnified when a radar is replaced in the workshop without specialised equipment due to a collision or sensor malfunction. In that case, mounting will introduce errors that are orders of magnitude greater than radar angle-finding algorithms, affecting system performance. Angular mounting inaccuracies (radar misalignment) can also occur during the standard lifetime of a vehicle due to wear of the mounting bracket or small collisions that could displace the sensor or bumper in front of it. Regulatory and automotive standards, such as ISO 26262, dictate that sensors must provide precise angles, and if that is not achievable, they should alert the system with a fault notification [12]. A system utilising a misaligned radar without being aware of the misalignment may act unpredictably (for instance, by detecting multiple non-existent objects). However, in practical scenarios, if the sensors operate in environments with slow-moving targets and a limited range, such as a parking lot, minor misalignment might not significantly impact system performance. Conversely, the most hazardous situations for the system and its occupants occur when the vehicle is moving quickly, as even a stationary object could generate ghost objects in the case of severely misaligned sensors.

This paper describes in detail the impact of radar misalignment on different aspects of AD/ADAS systems. In addition, it explains the methods of radar calibration with an emphasis on detecting and correcting angular mounting inaccuracies of a radar, commonly known as misalignment angles.

## 2. Influence of Radar Misalignment on Automotive Systems

### 2.1. Complete and Partial Sensor Blockage

**Figure 1:**
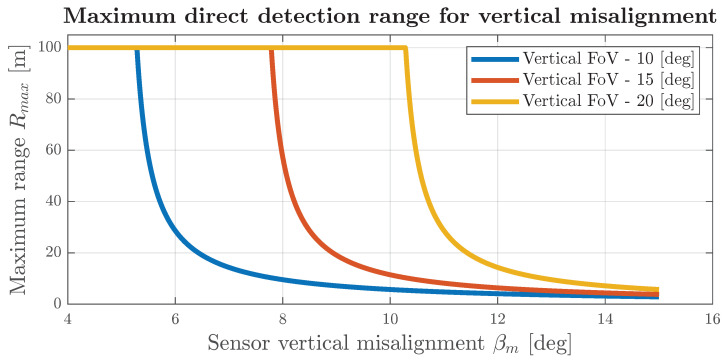
Maximum direct detection range for h = 0.5 m and $R_{FoV}$ = 100 m.

In cases where a radar is vertically misaligned so that all of the transmitted radar power is directed below the horizon line, the sensor can be partially or completely blinded. The radar is partially blinded when vertical misalignment still allows a part of the wave to be transmitted directly towards the target (the upper sensor field of view still encompasses the lower part of the targets). The maximum range of direct detection, in that case, can be calculated in a simplified form from trigonometric relations, and it is given by

(1) $$R_{max}=max\left(h\cdot \cot\left(-\frac{\beta_{FoV}}{2}+\beta_{m}\right),R_{FoV}\right),$$

where $R_{max}$ represents the maximum direct detection range [m] (distance from radar), *h* represents the sensor altitude (vertical mounting position above the ground) [m], $\beta_{FoV}$ represents the vertical field of view of the radar [deg], $\beta_{m}$ represents the elevation misalignment [deg], and $R_{FoV}$ represents the maximum radar range [m]. A comparison of the maximum direct path detection range is shown in Figure 1. It is observed that, above a certain elevation misalignment, in dependence on the FoV sensor, the degradation of the range becomes catastrophic and falls to values smaller than 20 m.

**Figure 2:**
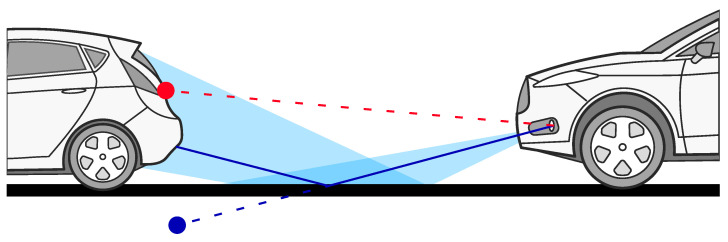
Sensor misaligned vertically towards the ground. The light blue area shows the radar transmitting power reflected from the ground. The dark blue line shows the reflected wave path (solid) and perceived wave path with detection as seen via the sensor (dashed). The red dashed line shows misaligned detection as perceived via the system, which is unaware of the misalignment.

It is also important to realise that a radar may be able to detect objects at greater distances than the one given in Equation (1) via an indirect path created from the reflection of the wave from the road. However, such detections will have a lower probability of occurrence as a result of only a fraction of the power reflected via the road. Furthermore, they will produce incorrect object elevation and range measurements. An example of such a scenario is illustrated in Figure 2. This phenomenon occurs due to the high reflectivity of most urban road surfaces at automotive radar frequencies. There have been multiple attempts to model the reflectivity of the road and the amount of power received from road reflection; however, the multitude of road materials and environmental conditions makes this a complex task. Louis Nagy in [13], designed a model capable of estimating the amplitude of backscattered road reflection for asphalt, brick and gravel roads. Environmental conditions have a huge impact on the reflectivity and backscatter of a road. A model that can predict the reflectivity of asphalt covered in ice or water was described and experimentally proven in [14]. However, not only the type of surface but also differences within the material structure will produce different results, as shown in the publication of measurements conducted by Kurz et al. in [15].

When a sensor is misaligned towards the sky, the target may not be visible to the radar sensor, as no surface could provide indirect reflection. The vehicle may approach a target that will be outside of the vertical field of view of the radar sensor and, depending on the amount of vertical misalignment, will provide reflections much later than in the case of an aligned sensor. In this subsection, consideration is given only to the problems related to the optical path to the target and outside the operational field of view. However, the detectability of an object within the expected field of view is lower the further the object is from the vertical centre of the FoV. This phenomenon is related to the gain patterns of the antennas, and it is explained in more detail in Section 2.2.

**Figure 3:**
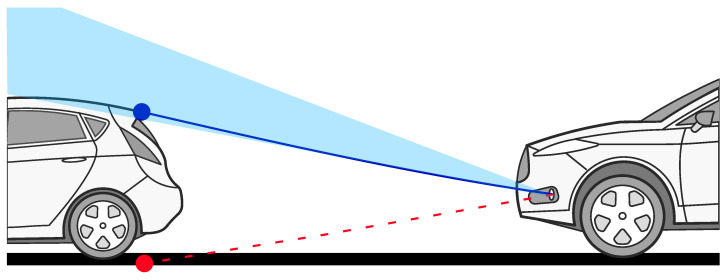
Sensor misaligned vertically towards the sky. The light blue area shows the radar transmitting power reflected from the ground. The dark blue line shows the radar wave path. The red dashed line shows misaligned detection as perceived via the system, which is unaware of the misalignment.

In the case of a radar with elevation measurement capabilities, another scenario must be taken into account. A radar misaligned to the sky, as in Figure 3, will observe detections from targets entering its vertical field of view as located below the radar, which may lead to an incorrect assumption from the system that an object can be driven over. The displacement of the detections as a direct consequence of the misalignment of the radar is described in Section 2.4.

A complete sensor blockage can occur when the radar is misaligned towards a non-transparent object that would completely block the radar wave, such as a component of the vehicle chassis, engine, etc. Both partial and complete blockages can significantly reduce radar observations and degrade their quality to the point where an algorithmic estimation of the misalignment angle based on radar detections is impossible. In such a case, sensor degradation should be detected via a specialised blockage algorithm, independent of radar detections, as mentioned in [10]. Blockages, severe misalignments, and measurement degradation should be considered as sensor faults and properly handled via the system to achieve ISO 26262 compliance [12].

### 2.2. Decrease in Maximum Range

A reliable estimation of the impact of misalignment on the signal amplitude can only be performed with detailed information about a sensor. In this subsection, calculations and analyses are performed based on antennas similar to those described in [8]. Similar steps could be used with different sensor models. Some radars with narrower elevation gain patterns that usually offer higher range capabilities will be even more susceptible to the relative range degradation described in this subsection.

Radars must be carefully designed for the purpose they serve. In the case of automotive sensors, they need to be small, be lightweight and have relatively low power to be considered fit for use in vehicles. Those requirements provide design challenges that enforce compromises in some of the radar specifications. One of the most important parameters of a radar is its maximum detection range. As described in [16], the maximum range of radar can be understood as the range at which the sensor should detect the objects it was designed for. This description may sound vague, but it is rooted directly in the received power ($G_{t}$) described by [16]:

(2) $$P_{r}=\frac{P_{t}G_{t}G_{r}\lambda^{2}\sigma_{RCS}}{{\left(4\pi \right)}^{3}R^{4}}.$$

The first part of the equation contains the components that are directly related to the radar—$P_{t}$ (transmitted power [W]), $G_{t}$ (transmission antenna gain [$\frac{1}{1}$]), $G_{r}$ (receiving antenna gain [$\frac{1}{1}$]), and λ (wavelength of the sensor [m]). Another part of the equation describes components that can vary based on the target—$\sigma_{RCS}$, the radar cross section [m^2^] (RCS), and *R*, the distance to the target [m]. In automotive scenarios, the RCS of targets can vary significantly between types of targets and exposition angles. Therefore, it is impossible to guarantee successful radar detection from some targets at the expected maximum radar range.

**Figure 4:**
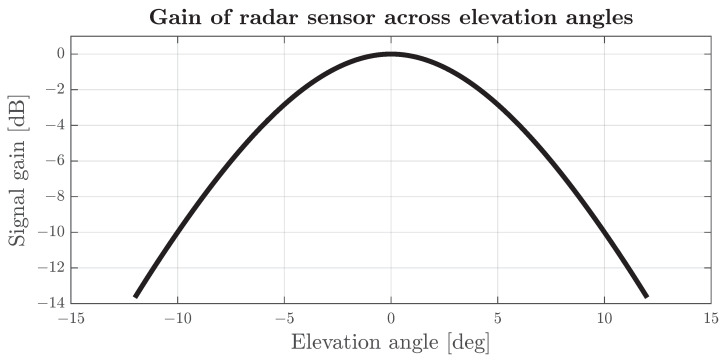
Difference between transmitted and received power caused by directional gain of antennas in elevation with the assumption that the transmitting and receiving antennas have the same mounting angles and positions and look at the same target (object/reflector).

Most automotive radars offer high azimuth coverage. Therefore, the impact on range due to horizontal misalignment is low. However, a relatively narrow elevation gain pattern in a vertical direction—essential to minimising unwanted, low-importance detections—may result in a significant gain decrease. As shown in Figure 4, a 10-degrees elevation misalignment, applied to the radar with approximate antenna parameters (both transmitting and receiving) from [8], results in a gain in signal power of approximately −10 dB.

**Figure 5:**
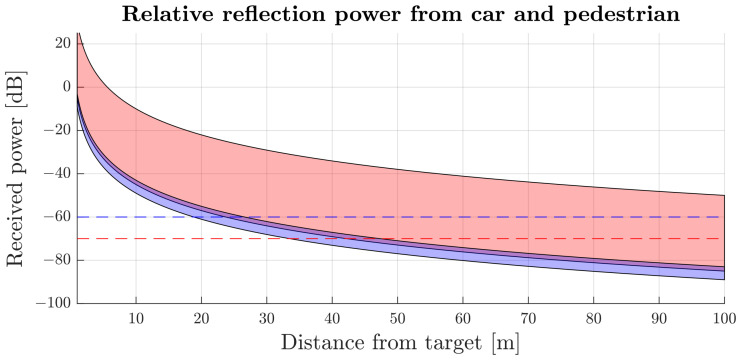
Received reflection power from a car (red area) and an adult pedestrian (blue area). The dashed lines depict the minimum power level required to achieve detection recognisable from background noise for a correctly mounted radar (red dashed line) and a radar misaligned by 10 degrees (blue dashed line).

In this study, the automotive radar target measurements by Geary et al. in [17] were used to examine two categories of targets: vehicles and pedestrians. Using the RCS values reported in [17], the reflection power relative to the transmitted power was calculated with Equation (2). The findings, depicted as the anticipated range of relative reflection power, are illustrated in Figure 5.

It can be assumed that, if the radar is properly aligned, and its nominal range is around 80 m, the receiver sensibility needs to be at a level of around −70 dB to detect vehicles at most exposition angles. With assumed baseline sensor sensitivity and the difference between received and transmitted power as a function of elevation shown in Figure 4, the misaligned sensor sensibility can be estimated, shifting the baseline to match the specific elevation misalignment. In the case of a sensor vertically misaligned by 10 degrees and a target in front of the vehicle or aligned sensor, but a target located at 10 degrees in elevation, the minimum power must be 10 dB higher than the baseline sensitivity to compensate for the lower transmitting and reception antenna gain.

Based on such analysis, sensors designed to provide radar detection for most vehicle classes in a range of 80 m can form the detection of an adult human male at 35–47 m, depending on the angle of exposure. Vertical misalignment of the radar by 10 degrees will affect the sensor’s maximum range for all targets. However, the most noticeable change affects pedestrian classes, for which the maximum range of an adult male’s detection should drop to 18–27 m. The impact on smaller types of pedestrians could be even more significant. To minimise the effects of such phenomena, the design of systems responsible for complex functions, such as autonomous driving, takes advantage of multiple types of sensors that observe the environment and ensure the safety of other road users. However, each sensor design should strive for maximum reliability to provide the best possible functionality.

### 2.3. Distinguishability of Targets from Clutter

Another important factor to consider when analysing the impact of radar misalignment on detection amplitude is related to the radar cross section of clutter present in the radar environment. Relevant targets must be distinguished from the surrounding clutter to provide useful information to the system [18]. As described in [19], the radar environment is populated with irrelevant objects for most radar applications. Those could be rocks, trees’ trunks or roots, irregular soil surfaces, and other objects. Depending on the terrain types, clutter can have different levels of amplitudes, depending on its characteristics. Some attempts were made to measure clutter for various terrains and radar frequencies. One of them is described in [20]. The measurements there resulted in attempts to provide mathematical methods to estimate the mean amplitude of the landscape clutter, such as [21]. An overview of different clutter models is presented in [22]; this work, among many other methods, presents a clutter model of the land for low grazing angles developed by the Georgia Institute of Technology [19]. This model provides a method for calculating the normalised reflectivity of the clutter $\sigma_{RCS}^{0}$, which denotes the RCS of the clutter $\sigma_{RCS}$ normalised according to the area of the surface illuminated by the radar pulse $A_{c}$. This relation is described in

(3) $$\sigma_{RCS}=\sigma_{RCS}^{0}A_{c}.$$

The area of an illuminated surface can be calculated based on the pulse beam shape factor $\alpha_{c}$, the range resolution of the radar, ρ, the slant range of the clutter, *r*, the azimuth beam width, $\theta_{az}$ and the grazing angle, $\phi_{gr}$, with

(4) $$A_{c}=\alpha_{c}\rho r\theta_{az}\sec\phi_{gr}.$$

The grazing angle is calculated as

(5) $$\phi_{gr}=\arcsin\left(\frac{h}{r}+\frac{h^{2}}{2R_{e}r}-\frac{r}{2R_{e}}\right),$$

where *h* denotes the mounting height of the radar and $R_{e}$ the Earth’s radius, but for most automotive use cases, where a radar’s range is much smaller than 1 [km], the equation can be simplified to

(6) $$\phi_{gr}=\arcsin\left(\frac{h}{r}\right).$$

Finally, normalized clutter reflectivity can be calculated with

(7) $$\sigma_{RCS}^{0}=A{\left(\psi +C\right)}^{B}\exp\left[\frac{-D}{1+\frac{0.1\sigma_{h}}{\lambda }}\right].$$

**Figure 6:**
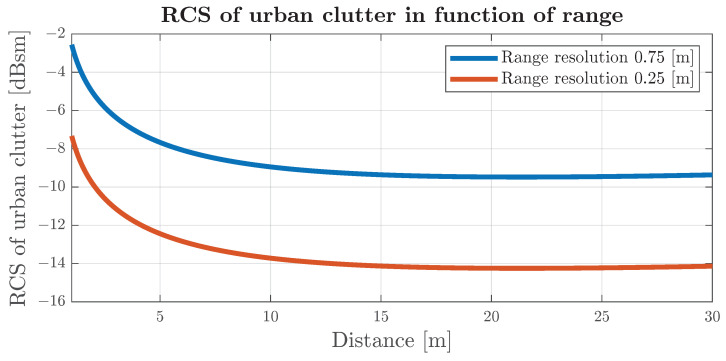
Calculated values of mean urban clutter for radars with a range resolution of 0.75 m and 0.25 m. Radar parameters used: height above ground (*h*)—0.4 m, azimuth beam width ($\theta_{az}$)—90 degrees, beam shape factor ($\alpha_{c}$)—1 and radar frequency—15 GHz.

*A*, *B*, *C* and *D* are model parameters available in Table 1, ψ is the depression angle, $\sigma_{h}$ is the standard deviation of surface roughness in [cm] and λ is the radar wavelength. Equations (3)–(5) and (7), as well as Table 1, can be used to estimate the RCS of urban clutter as a function of distance. The results of the calculation are presented in Figure 6.

The distribution of the radar clutter amplitude for low grazing angles forms the Weibull distribution [19,22,23]. Depending on the type of environment and radar frequency, the parameters of a distribution may form a long tail to cross sections much higher than the average. A detailed guide to modelling the clutter distribution for low grazing angles is presented in [24]. The referenced models are designed for frequencies up to X-band (8.0–12.0 GHz), which would not be sufficient for modern automotive radars that operate on 23–24 GHz [25,26,27] and 77–81 GHz [28,29,30,31,32] frequencies, indicating the promising area for research on clutter modelling for those sensors.

Figure 6 shows the estimated mean value of the clutter RCS, which can be compared with the RCS of adult pedestrians from [17], which is in the range of −2 to −9 dBsm. For radar that is misaligned towards the ground and observes clutter with a higher antenna gain, a pedestrian may generate reflections that may be impossible to classify as a relevant target (object).

### 2.4. Displacement of the Detections and Loss of Coverage in Areas of Interest

The displacement of the detections is a direct consequence of radar misalignment. The reported azimuths and elevations contain systematic errors; i.e., they are biased due to a constant shift across all of the measurements

(8) $$\left\{\begin{array}{c} {\hat{\alpha }}_{n}=\alpha_{n}+\alpha_{m}\\ {\hat{\beta }}_{n}=\beta_{n}+\beta_{m} \end{array}\right.,$$

where ${\hat{\alpha }}_{n}$ is the corrected azimuth measurement of the *n*-th radar detection, $\alpha_{n}$ is the azimuth measurement of the *n*-th radar detection, $\alpha_{m}$ is the azimuth misalignment, ${\hat{\beta }}_{n}$ is the corrected elevation measurement of the *n*-th radar detection and $\beta_{n}$ is the elevation measurement of the *n*-th radar detection.

The displacement described in Equation (8) in 3D cartesian coordinates can take a form of rotation that can be described with matrix equation

(9) $$\left[\begin{array}{c} {\hat{x}}_{n}\\ {\hat{y}}_{n}\\ {\hat{z}}_{n} \end{array}\right]=R_{z}R_{y}R_{x}\left[\begin{array}{c} x_{n}\\ y_{n}\\ z_{n} \end{array}\right],$$

where ${\hat{x}}_{n}$ and $x_{n}$ denote, respectively, the corrected and measured longitudinal positions of detection, ${\hat{y}}_{n}$ and $y_{n}$ denote the lateral position of detection, and ${\hat{z}}_{n}$ and $z_{n}$ denote the vertical position of detection. $R_{x}$, $R_{y}$ and $R_{z}$ are rotation matrices along the respective axes.

**Figure 7:**
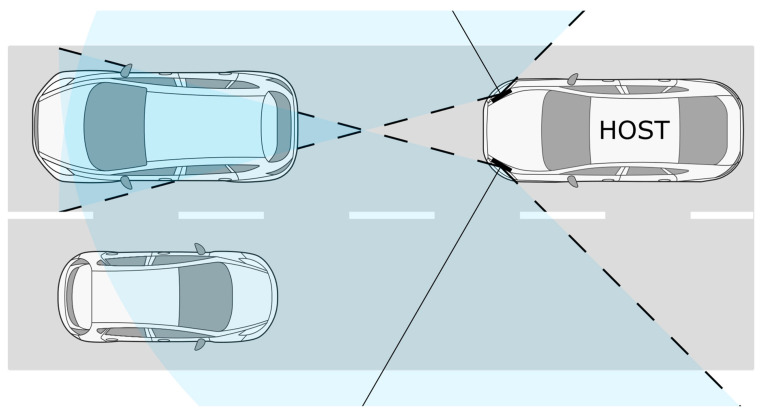
Potential target line misclassification scenario and visualization of the front radar blind spot with sensors’ FoVs coverage (blue area), FoVs borders (dashed lines) and sensor horizon angles (solid lines).

In the case of the common road scenario depicted in Figure 7, misalignment of the frontal radars may result in the incorrect classification of the road lanes alongside which road targets are moving. When assuming a straight road and a point-based target precisely in the middle of the road lane, the minimum misalignment sufficient to incorrectly classify the target road lane can be given as a function of distance from the radar

(10) $$\alpha_{m}^{L}=\arctan\left(\frac{W_{R}}{2r}\right).$$

**Figure 8:**
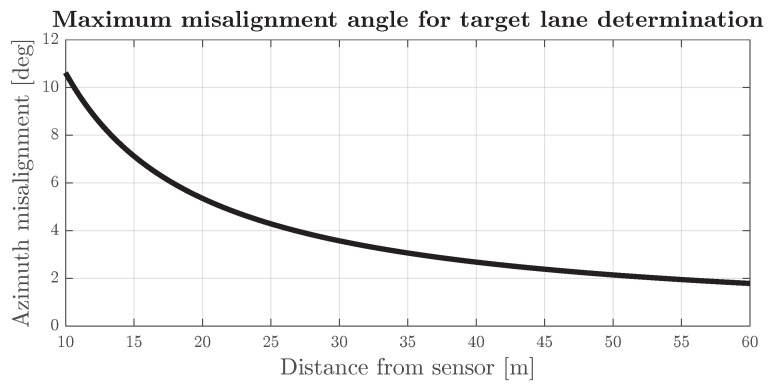
Misalignment angle that would cause a point-based target to be misplaced on an incorrect road lane in the function of distance.

The azimuth misalignment sufficient to incorrectly classify the detection is denoted as $\alpha_{m}^{L}$, $W_{R}$ is the width of the road lane and *r* is the distance between the radar and the detected object (detection range). The resulting function is presented in Figure 8 with the assumption that the road lane has a standard width of 3.75 m.

Misalignment can also impact radar coverage in areas of interest. In the case of a system built with corner sensors, as shown in Figure 7, depending on the boresight angle of those radars, the system has a blind spot directly in front of the vehicle. In its simplest form, it can be described as a triangular area between sensors with a bisector length that depends on the boresight angle and the horizontal field of view of the sensor. Although active safety systems also include frontal radar or a camera that covers this area, it may be detrimental to the system’s performance if the size of a blind spot of corner radars is excessively increased.

Both frontal corner and rear corner radars may be misaligned outward (for front left sensor counterclockwise; for front right sensor clockwise). In such a scenario, the blind spot between these two sectors will become bigger, and the magnitude of its impact will depend on the system design. The bisector length of the area that is not covered by the field of view of frontal sensors, as shown in Figure 7, can be derived from trigonometric relations, and it is given in

(11) $$B_{L}=\cot\left(\frac{\alpha_{FoV}}{2}-\alpha_{b}+\alpha_{m}\right)\cdot W_{S}.$$

**Figure 9:**
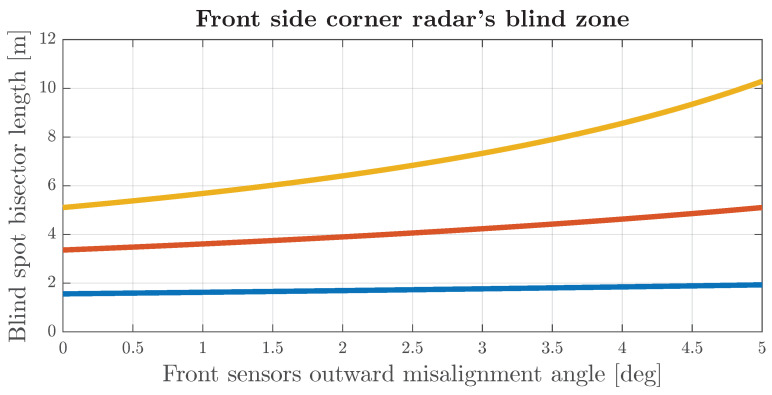
Blind spot bisection length for mounting angles of 45 degrees (blue), 60 degrees (orange) and 65 degrees (gold).

The bisector length in Equation (11) is denoted as $B_{L}$, $\alpha_{FoV}$ is the horizontal field of view of the radar, $\alpha_{b}$ is the absolute boresight angle, $\alpha_{m}$ is the azimuth misalignment and $W_{S}$ is the distance between those sensors. Figure 9 shows the impact on the size of the blind spot bisector for different mounting angles of frontal radars with the assumption that the distance between radars is equal to 1.8 m and the horizontal sensor field of view is equal to 150 degrees.

### 2.5. Inaccuracies of Target Velocity Vector

An essential feature of automotive radars is their ability to determine the direction and velocity of surrounding objects. Depending on the available computing resources and system architecture, this feature can be realised based on the phase difference between multiple detections of the same object [33] or by using information fusion when multiple radars cover the same area [34]. A simpler approach to finding the components of the velocity vector of the target is based on the radar range-rate equation [35], using multiple detections measured from one target, described in

(12) $${\dot{r}}_{n}=\left(T_{x}-V_{x}\right)\cos\alpha_{n_{\mathrm{VCS}}}+\left(T_{y}-V_{y}\right)\sin\alpha_{n_{\mathrm{VCS}}},$$

where $T_{x}$ and $T_{y}$ are target velocity components in a vehicle coordinate system, $V_{x}$ and $V_{y}$ are the host vehicle velocity components in the longitudinal and lateral axes, respectively, $\alpha_{n_{\mathrm{VCS}}}$ is an angle at which *n*-th detection is observed to be aligned in a vehicle coordinate system (an angle equal to 0 points to the same direction as a longitudinal axis of the host vehicle, which is also the direction of the x-axis of the vehicle coordinate system), and ${\dot{r}}_{n}$ is the range rate (Doppler velocity, i.e., the velocity component of an observed point projected on the direction towards the sensor mounting location) of *n*-th detection.

Equation (12) is a projection of the relative velocity of the target on a directional vector of observed detection. Another representation of Equation (12), which better represents the error distribution, takes the form of a matrix or a complex equation that can be described in the following relations:

(13) $$\alpha_{n_{\mathrm{VCS}}}=\alpha_{n}+\alpha_{b}+\alpha_{m},$$

(14) $$\bar{R}=\left(\bar{T}-\bar{V}\right)\bar{D_{n}^{*}},$$

(15) $${\dot{r}}_{n}=ℜ\left\{\bar{R}\right\},$$

(16) $$\bar{D_{n}^{*}}=\cos\alpha_{n_{\mathrm{VCS}}}-\hat{i}\sin\alpha_{n_{\mathrm{VCS}}},$$

(17) $$\bar{D_{n}}\cdot \bar{D_{n}^{*}}=1.$$

where $\bar{T}$ and $\bar{V}$ are complex representations of target velocity vectors and host velocity vectors, and $\bar{D_{n}^{*}}$ is a complex conjugate of detection’s directional vector ($\bar{D_{n}}$). Equation (14), therefore, represents a rotation of velocity vectors at an angle that would make the directional vector of detection reside entirely in the real domain, satisfying Equation (17), and $\bar{R}$ represents the relative velocity vector rotated to satisfy Equation (15). The complex representation in Equation (16) and the fact that $\alpha_{n_{\mathrm{VCS}}}$ can be decomposed with (13) are used to derive

(18) $$\bar{D_{n}^{*}}=\left(\cos\alpha_{n}-\hat{i}\sin\alpha_{n}\right)\cdot \left(\cos\alpha_{b}-\hat{i}\sin\alpha_{b}\right)\cdot \left(\cos\alpha_{m}-\hat{i}\sin\alpha_{m}\right).$$

The decomposed complex numbers in Equation (18) are purely rotational (have a unit magnitude). Therefore, their influence affects only the phase (the angle of the relative velocity vector), which can be proven by comparing the true relative velocity vector ($\bar{R_{T}}$) to the misaligned relative velocity vector ($\bar{R_{M}}$).

(19) $$\left\{\begin{array}{c} \bar{R_{T}}=\left(\bar{T_{T}}-\bar{V}\right)\bar{D_{n}^{*}}=\bar{X_{T}}\bar{D_{n}^{*}}\\ \bar{R_{M}}=\left(\bar{T_{M}}-\bar{V}\right)\bar{D_{n}^{*}}e^{j\alpha_{m}}=\bar{X_{M}}\bar{D_{n}^{*}}e^{j\alpha_{m}}\\ ℜ\left\{\bar{R_{T}}\right\}=ℜ\left\{\bar{R_{M}}\right\}={\dot{r}}_{n} \end{array}\right.$$

To achieve this, the system of equations presented in Equation (19) must be solved. To shorten the following equations, the initial relative velocity vectors ($\bar{X}$), before rotation via $\bar{D_{n}^{*}}$, are introduced into the equation. The property of complex numbers is then used to represent the real part of a complex number with the number and its conjugate, resulting in

(20) $$\begin{array}{cc} & \frac{\bar{X_{M}}\bar{D_{n}^{*}}e^{j\alpha_{m}}+\bar{X_{M}^{*}}\bar{D_{n}}e^{-j\alpha_{m}}}{2}=\frac{\bar{X_{T}}\bar{D_{n}^{*}}+\bar{X_{T}^{*}}\bar{D_{n}}}{2}\\ & \frac{\bar{D_{n}^{*}}\left(\bar{X_{M}}e^{j\alpha_{m}}-\bar{X_{T}}\right)+\bar{D_{n}}\left(\bar{X_{M}^{*}}e^{-j\alpha_{m}}-\bar{X_{T}^{*}}\right)}{2}=0. \end{array}$$

After the cases in which Equation (20) has an infinite number of solutions (such as the case where the relative velocity magnitude is equal to zero) are removed from consideration, the only solution left is given by

(21) $$\bar{X_{M}}=\bar{X_{T}}e^{-j\alpha_{m}}.$$

This relation can then be used to calculate the relation between those true and misaligned vectors $\bar{R_{T}}$ and $\bar{R_{M}}$.

(22) $$\frac{\bar{R_{T}}}{\bar{R_{M}}}=\frac{\left(\bar{T_{T}}-\bar{V}\right)\bar{D_{n}^{*}}}{\left({\bar{T}}_{T}-\bar{V}\right)\bar{D_{n}^{*}}e^{-j\alpha_{m}}}=e^{j\alpha_{m}}$$

Equation (22) provides a transformation vector that can be used to correct the relative velocity vector that was calculated without knowledge of the misalignment angle to the true value. It is important to note that $\alpha_{n_{\mathrm{VCS}}}$, denoted in Equation (13), is the true value of the azimuth. Therefore, the misaligned value can be obtained by subtracting $\alpha_{m}$ from it. A misaligned $\bar{D_{n}^{*}}$ can be obtained via multiplication with $e^{j\alpha_{m}}$, which removes the $\alpha_{m}$ component from Equation (18).

(23) $$\left\{\begin{array}{c} \begin{array}{cc} & R_{x}=\frac{{\dot{r}}_{1}\sin\alpha_{2_{\mathrm{VCS}}}-{\dot{r}}_{2}\sin\alpha_{1_{\mathrm{VCS}}}}{\cos\alpha_{1_{\mathrm{VCS}}}\sin\alpha_{2_{\mathrm{VCS}}}-\cos\alpha_{2_{\mathrm{VCS}}}\sin\alpha_{1_{\mathrm{VCS}}}}\\ & R_{y}=-\frac{{\dot{r}}_{1}\cos\alpha_{2_{\mathrm{VCS}}}-{\dot{r}}_{2}\cos\alpha_{1_{\mathrm{VCS}}}}{\cos\alpha_{1_{\mathrm{VCS}}}\sin\alpha_{2_{\mathrm{VCS}}}-\cos\alpha_{2_{\mathrm{VCS}}}\sin\alpha_{1_{\mathrm{VCS}}}} \end{array} \end{array}\right.$$

(24) $$\frac{R_{x_{T}}+\hat{i}R_{y_{T}}}{R_{x_{M}}+\hat{i}R_{y_{M}}}=\cos\alpha_{m}+\hat{i}\sin\alpha_{m}=e^{j\alpha_{m}}$$

The same relation can be proven by calculating relative velocity components using information from two radar detections in Equation (23) for true and misaligned angles and comparing them (24).

The error of relative velocity heading can be concluded based on Equations (22) and (24) and assuming that the Gaussian distribution of the radar’s azimuth error can be denoted with

(25) $$E\left(\arg\bar{R}\right)=N\left(-\alpha_{m},\sigma_{R}^{2}\right).$$

The random component of the heading angle error ($\sigma_{R}$) is caused by azimuth measurement errors and may be influenced by the spread of detections and their number. It can be minimised with an increase in the resolution and angle separation of the radar, which would provide a high amount of radar detections per single object. Further simulations assume that an estimation caused by imperfections in radar detections is negligible (detections are ideal).

To find the inaccuracy of the target’s velocity vector, Equation (14) can be transformed into a form derived in

(26) $$\begin{array}{cc} \frac{\bar{R_{T}}}{\bar{R_{M}}} & =e^{j\alpha_{m}}\\ \bar{R_{M}} & =\bar{R_{T}}e^{-j\alpha_{m}}\\ \bar{T}\bar{D_{n}^{*}}\bar{D_{n}} & =\bar{R}\bar{D_{n}}+\bar{V}\bar{D_{n}^{*}}\bar{D_{n}}\\ \bar{T_{M}} & =\bar{R}\bar{D_{n}}e^{-j\alpha_{m}}+\bar{V}. \end{array}$$

The resulting misaligned target velocity, $\bar{T_{M}}$, can be divided by the true target velocity, $\bar{T_{T}}$, in the following derivation:

(27) $$\begin{array}{cc} \frac{\bar{T_{T}}}{\bar{T_{M}}} & =\frac{\bar{R}\bar{D_{n}}+\bar{V}}{\bar{R}\bar{D_{n}}e^{-j\alpha_{m}}+\bar{V}}\\ \frac{\bar{T_{M}}}{\bar{T_{T}}} & =\frac{\bar{R}\bar{D_{n}}e^{-j\alpha_{m}}+\bar{V}}{\bar{R}\bar{D_{n}}+\bar{V}}\\ \frac{\bar{T_{M}}}{\bar{T_{T}}}+\frac{\bar{V}e^{-j\alpha_{m}}}{\bar{R}\bar{D_{n}}+\bar{V}} & =\frac{\bar{R}\bar{D_{n}}e^{-j\alpha_{m}}+\bar{V}}{\bar{R}\bar{D_{n}}+\bar{V}}+\frac{\bar{V}e^{-j\alpha_{m}}}{\bar{R}\bar{D_{n}}+\bar{V}}\\ \frac{\bar{T_{M}}}{\bar{T_{T}}}+\frac{\bar{V}e^{-j\alpha_{m}}}{\bar{R}\bar{D_{n}}+\bar{V}} & =\frac{\left(\bar{R}\bar{D_{n}}+\bar{V}\right)e^{-j\alpha_{m}}+\bar{V}}{\bar{R}\bar{D_{n}}+\bar{V}}\\ \frac{\bar{T_{M}}}{\bar{T_{T}}} & =e^{-j\alpha_{m}}-\frac{\bar{V}e^{-j\alpha_{m}}}{\bar{R}\bar{D_{n}}+\bar{V}}+\frac{\bar{V}}{\bar{R}\bar{D_{n}}+\bar{V}}\\ \frac{\bar{T_{M}}}{\bar{T_{T}}} & =\frac{1+\frac{∥V∥}{∥T∥}e^{j(∠V-∠T)}\left(e^{j\alpha_{m}}-1\right)}{e^{j\alpha_{m}}}\\ \frac{\bar{T_{T}}}{\bar{T_{M}}} & =\frac{e^{j\alpha_{m}}}{1+\frac{∥V∥}{∥T∥}e^{j(∠V-∠T)}\left(e^{j\alpha_{m}}-1\right)}. \end{array}$$

**Figure 10:**
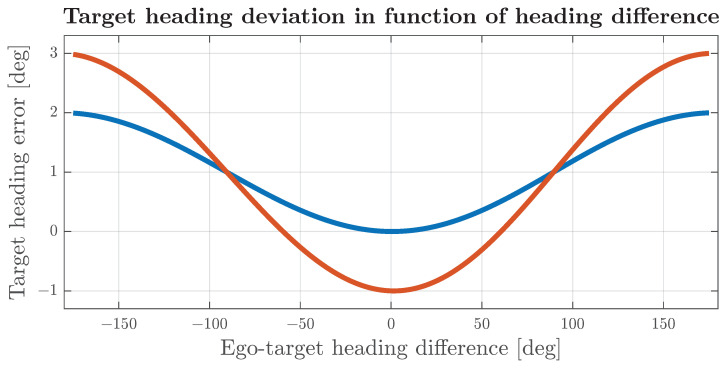
Influence of misalignment angle of 1 degree on target heading error for target speed equal to ego speed (blue) and ego vehicle speed two times greater than the target (red).

**Figure 11:**
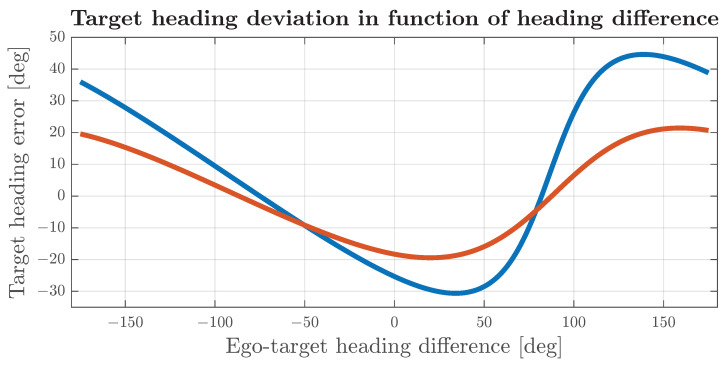
Influence of misalignment angle of 7 degrees on target heading error for target speed five times greater than ego speed (blue) and misalignment of 1 degree for ego vehicle speed twenty times greater than the target (red).

The impact of misalignment on the target velocity that can be observed in Equation (27) influences both the magnitude and the angle of the target velocity vector. In cases where the ego vehicle is stationary or moving much slower than the target vehicle, the heading error and magnitude error will be close to zero. In a case where the target velocity is within the same order as the ego vehicle velocity, and the misalignment angle is less than 5 degrees, the impact on the heading can be approximated to a constant component equal to $\alpha_{m}$ and a sinusoidal component with a magnitude close to $\frac{∥V∥}{∥T∥}\alpha_{m}$, dependent on the difference between the vehicle and target heading. The simulation results that confirm such behaviour are shown in Figure 10. In cases where the magnitude of the velocity of the ego is much greater than the target velocity or the misalignment angle is significantly greater than 5 degrees, the error of the heading angle is affected by strong non-linearity, as depicted in Figure 11, and cannot be intuitively related to $\alpha_{m}$.

The varying bias of heading errors caused by the misalignment angle $\alpha_{m}$ without proper estimation methods and compensation exerts a serious, non-linear influence on the estimated target heading and speed. It is worth mentioning that the plausibility of the estimated target heading can be further verified, for example, by comparison to an estimated orientation such as that described in [36,37,38,39]. However, those methods, on top of the estimation error, are also prone to misalignment in the same way as $\bar{R}$—due to an unknown misalignment that affects the azimuth angle, as in Equation (13).

### 2.6. Data Association Problems

One of the most important functionalities of modern, active safety systems is the fusion of information from multiple sensors and the tracking of the vehicle surroundings. An adequately designed method, consisting of different sensor types with other pros and cons, can robustly estimate the environment and provide object information that is not feasible for one sensor. An example of a multisensor fusion is a system composed of a camera, radar and lidar observing the same field of view. In such a design, the radar provides the accurate relative velocity of the target, the lidar provides the high-resolution shape and precise position and the camera provides additional information that can help determine the class of a target (object) and improve the detection of small and non-reflective road elements like traffic lights or road lane markers. An example of the camera, radar and lidar fusion used to control the car is described in [40], where complex neural network architecture combines data from sensors to control the steering angle, throttle and brakes. As described in [41], AD can be implemented in an end-to-end approach (with a complex neural network) or in a modular approach, in which data tracking and fusion are performed using algorithms or smaller neural networks with clearly defined goals. The algorithmic approach to data fusion with various available methods is extensively described in [42].

One of the challenges of data fusion methods operating in an environment with an unknown number of targets is determining which tracked object it belongs to (association of track/measurement to existing object) or whether it should form an entirely new object hypothesis (spawning a new track or splitting an existing object) [43]. One of the methods that can associate measurements with tracked objects is joint probabilistic data association (JPDA) and its variations described in [44,45,46,47,48,49]. When the data cannot be associated with any existing object, they need to be stored as an object hypothesis for the purpose of further data association from other sensors or the next radar scan. To filter out objects that may not exist from the relevant target-tracking and fusion algorithms, one can implement the estimation of existence likelihood/probability [50,51,52,53,54]. Intuitively, it can be explained that, in each case where any sensor detects an object with expected states that allow for association, the existence likelihood is increased. On the other hand, each time the system cannot associate any measurement to an object, its existence likelihood is decreased. Even though the system may track a maximum number of objects that are within allowed computational limits, the lifecycle of an object visible to other algorithms begins when its existence likelihood passes a certain threshold and ends when it drops below another.

Bar-Shalom describes a test for determining the association of objects based on a squared Mahalanobis distance of state differences [42]. The formula for an estimated state difference (${\hat{\Delta }}^{of}$) of a measured object (${\hat{x}}^{o}$) and a fusion-based object (${\hat{x}}^{f}$) at time index *k* is given in

(28) $${\hat{\Delta }}^{of}\left(k\right)={\hat{x}}^{o}\left(k\right)-{\hat{x}}^{f}\left(k\right).$$

When assuming an error independence covariance matrix of state differences ($P^{of}$) will take a form given by

(29) $$P^{of}\left(k\right)=P^{o}\left(k\right)+P^{f}\left(k\right),$$

where $P^{o}$ denotes the covariance of a measured object, and $P^{f}$ denotes the covariance of a fused object.

The results of calculation from Equations (28) and (29) can be used to calculate the squared Mahalanobis distance and compare it with the preselected threshold $D_{\alpha }$ to determine whether a measured object can be associated with the fused object as in

(30) $$D\overset{\Delta }{=}{\hat{\Delta }}^{of}{\left(k\right)}^{T}{\left[P^{of}\left(k\right)\right]}^{-1}{\hat{\Delta }}^{of}\left(k\right)<D_{\alpha }.$$

In this subsection, a system based on the object generated via the velocity estimation method described in Section 2.5 demonstrates the behaviour of data association. Let us assume that the system receives an ideal fused object and attempts to associate the estimated object with it. The estimated object is created based on 16 detections generated every 2 degrees. Then, Gaussian noise is applied to the azimuth measurement and range-rate measurement. The algorithm selects three random detections and uses Equation (23) to calculate the estimated target velocity and heading; then, the process is repeated 16 times, and outputs are collected. The median of acquired headings is used as a final estimate. The estimated heading angle will be compared with the heading of the fused object; if the difference is greater than two standard deviations from the heading, the estimated object will not be associated, and if the same situation occurs for four consecutive scans, the fused object should be split, and a new track should be formed. Such parameters were selected with the assumption that, if errors in heading estimates follow a Gaussian distribution, and the radar performs 20 measurements per second, then an incorrect track split will occur less than once every 3 h. The simulation generates relatively wide targets that span 30 degrees of the azimuth FoV to minimise the influences of other phenomena on the system. Both the target and ego-vehicle velocity vectors move with a constant velocity—the target moves south, and the ego vehicle moves north. Due to the wide target, azimuth misalignment will not influence the association in terms of the location of the fused object and measurement because the generated detections will at least partially intersect with the fused object. In this scenario, the heading of the estimated target is the main factor that contributes to incorrect object splitting.

**Figure 12:**
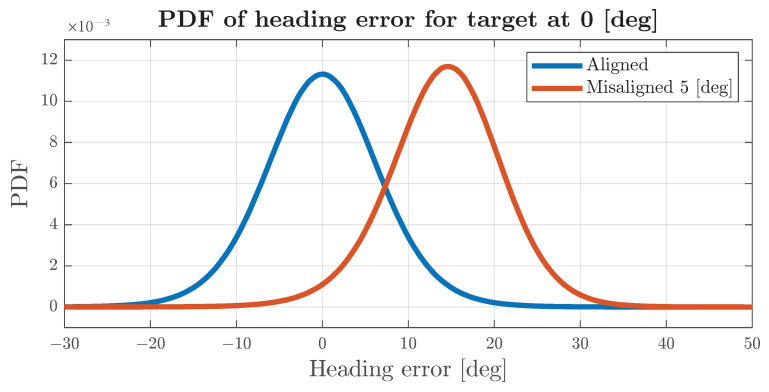
Probability density function of frontal target heading error for aligned and misaligned radar based on simulation results. Parameters used for Monte Carlo simulation: standard deviation of $\alpha_{n}$—2 degrees, standard deviation of ${\dot{r}}_{n}$—0.5 m/s, target azimuth spread—30 degrees, T—$10e^{j\pi }$ m/s, V—$20e^{j0}$ m/s, number of samples—$10^{8}$.

**Figure 13:**
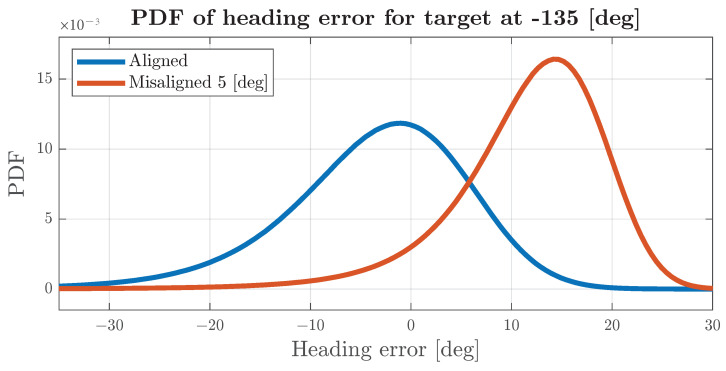
Probability density function of target heading error for the aligned and misaligned radar based on simulation results. Simulation inputs are the same as for Figure 12, except the target center location at $\alpha_{\mathrm{VCS}}=-135$ degrees.

Figure 12 shows the error distribution for a target in front of the vehicle. For a properly aligned radar, the standard deviation of the frontal target heading is below 7 degrees and the mean is close to zero. In cases where the target is located in other azimuths, due to azimuth and range-rate errors passing through a non-linear model, the error distribution changes shape, and even for aligned radars, it is not a zero-mean, as shown in Figure 13. Differences in heading-error distributions suggest that, for targets at different azimuth angles, it may be beneficial to select different association thresholds, which will maintain the association probability within the same range as for frontal targets. The simulation results include the calculation of separate thresholds for targets at different locations.

**Figure 14:**
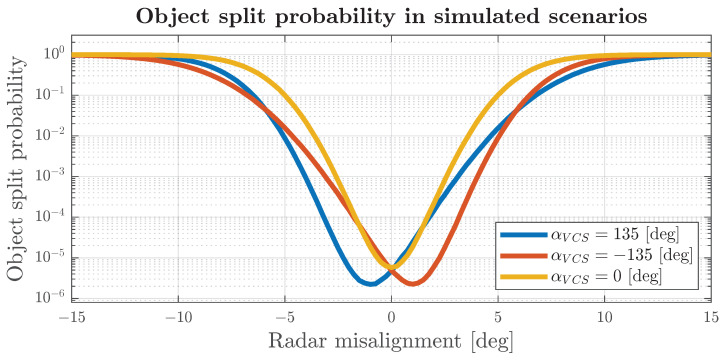
Probability of unwanted object splitting in the simulated scenario for three locations of target centre $\alpha_{\mathrm{VCS}}$ in a vehicle coordinate system.

Three scenarios were implemented for a target at −135, 0 and 135 degrees with respect to the front of the vehicle to visualise the impact of radar misalignment on undesired object splitting. A distinct threshold was determined for each scenario based on the target error distribution. Subsequently, for radar misalignment angles ranging from −15 to 15 degrees in increments of 0.25 degree, calculations were carried out on $10^{6}$ radar scans, using the aforementioned steps. The split probability for each of these steps is presented in Figure 14. Biases for non-zero target angles may cancel themselves when the radar is misaligned at specific angles. Those biases can be compensated for in more complex heading-estimation algorithms. However, it will not mitigate the impact of an unknown radar misalignment. As radar misalignment increases, the number of false track splits increases dramatically. In our simulated environment, instead of appearing on average once every 3 h for an aligned system, it will appear once per 45 min due to 1-degree misalignment, once in four minutes for 2-degrees misalignment and three times per minute for 3-degrees misalignment. At 5-degrees misalignment, the system will most likely become nonoperational due to the massive amount of non-existent objects reported via the system.

### 2.7. Localisation and Mapping

Errors in antenna alignment can also affect Simultaneous Localisation and Mapping (SLAM) algorithms. SLAM aims to continuously update the environment map generated from the reflections of fixed objects, all translated into the Global Coordinate System (GCS). For detections in the GCS, precise vehicle dynamics and radar measurements are necessary. If the radar is misaligned, the conversion of detections from the Sensor Coordinate System (SCS) to the GCS will be subject to a systematic error [55]. This error, introduced into the position of a stationary target observed at range *r*, from a vehicle with an orientation relative to GCS (φ), will be described in

(31) $$\left\{\begin{array}{c} \Delta_{x_{n}}=r\cos\left(\alpha_{b}+\alpha_{n}+\alpha_{m}+\varphi \right)-r\cos\left(\alpha_{b}+\alpha_{n}+\varphi \right)\\ \Delta_{y_{n}}=r\sin\left(\alpha_{b}+\alpha_{n}+\alpha_{m}+\varphi \right)-r\sin\left(\alpha_{b}+\alpha_{n}+\varphi \right) \end{array}\right..$$

The deviation outlined in Equation (31) will deteriorate the quality of the collected feature map, blurring the image of the environment.

Using SLAM or Integrated Sensing and Communication (ISAC) [56] for localisation can lead to orientation inaccuracies due to misalignment. The estimation of a vehicle state using a single sensor will be influenced by systematic errors that counter the misalignment of the radar. Conversely, a system utilising multiple sensors is expected to be more resilient to misalignment and enhance localisation capabilities, assuming that sensors are misaligned by a random zero-mean distribution.

According to [56], the ISAC radar system consists of the base station, transmitting and receiving antenna arrays, and sensors with antenna arrays capable of both sensing and communication. This configuration leverages a significant amount of input for precise localisation, allowing it to determine the user’s relative position with great accuracy. However, if the base station antenna is misaligned, the calculation of the user’s position in GCS will suffer from the same error, as outlined in Equation (31). Consequently, when ISAC is considered to replace other user localisation systems, taking into account algorithmic antennas’ alignment may be necessary to achieve high-resolution positioning in GCS.

## 3. Physical Correction Methods

The impact of radar misalignment, in addition to the easy-to-compensate algorithmically angular errors, impacts the amplitude of relevant detections, and it can amplify the clutter amplitude and reduce the radar detection range significantly for classes that are challenging even with a perfectly aligned radar. Those effects can be nullified only by correcting the antenna beam direction back to the optimal position, with a sensor housing capable of applying corrections to mounted radars. It may be possible to use external sensors to estimate radar misalignment online and use a motor to align radars, as proposed by Pinnock [57]. Such a method could physically minimise radar misalignment. However, it may be too expensive for mass production. Other methods proposed by inventors have focused on an adjustable bracket design that can be corrected with a specified angle once the misalignment is measured or estimated [58,59,60,61,62,63]. The lack of precise actuators significantly reduces costs. However, it requires additional mechanical components, and it may be debatable whether car manufacturers will find these methods attractive in the highly competitive market.

## 4. Algorithmic Correction Methods

Murad et al. describe short scenarios where alignment should be performed in [10,11]. According to this description, algorithms can be classified based on the use-case scenario. The mentioned cases include end-of-line alignment (EOL), in which radars are aligned at the end of the vehicle assembly to minimise production process inaccuracies and control quality. A vehicle workshop is the second use case in which a trained technician may perform the calibration process after replacing radars or conducting vehicle repairs. The third case mentioned is online alignment, in which the radar continuously monitors and corrects misalignment errors. Two not-described use cases may be worth mentioning: calibration conducted by the untrained driver, guided by simple instructions (in the case where there is a suspicion that the radar could be displaced in an accident, and the vehicle workshop is not available) and the estimation of the misalignment angle for offline analysis (such as finding out the true misalignment angle in a long dataset).

This paper attempts to clarify algorithm types with an additional classification system based on the required setup and initial assumptions. The alignment would fall into further described classes: static alignment, for methods that require a specific calibration setup that may need to be connected to the vehicle to determine misalignment angles; track alignment, for techniques that require particular road targets or road shapes to estimate angle, but whose radar or vehicle ECU performs a calibration process during the drive on track; dynamic alignment, for algorithms that monitor misalignment angles during a vehicle’s lifetime without supervision; and post-factum alignment, for methods that estimate the misalignment angle based on a large recorded dataset from the radar after a test drive.

The computational complexity, RAM consumption and ROM consumption will determine whether the alignment algorithms can be executed via the sensor processor or vehicle ECU, which is necessary for track and dynamic alignments. They will also differ according to the number of radars required for operation. Algorithms will also vary regarding the expected time required for an accurate assumption and its final accuracy.

### 4.1. Static Alignment

Algorithms in this category can be separated into two use cases: EOL alignment—performed at the end of the vehicle production process, and service alignment—used in the vehicle workshop after radar replacement or repairs near the sensor [64]. Both of these use cases have specific requirements. In the case of EOL, there is usually a limited number of assembly lines, which enables the use of more expensive calibration equipment. However, the area where calibration is performed is typically limited and shared with other equipment, which limits the use of methods that require multiple targets or targets at a greater distance. As vehicle production increases, car manufacturers also seek ways that will take as little time as possible to reduce the risk that calibration becomes a production bottleneck. On the other hand, it should be possible to perform service alignment in almost all certified workshops. This implies that the method should not rely on expensive devices but can also take advantage of the fact that the calibration process may take a bit longer without a significant impact.

The radar’s horizontal angle may be aligned, placing at least one reflective target at a predetermined location relative to the radar. The radar may then calculate the azimuth deviation between the measured and expected angles to determine the azimuth misalignment [63,65]. Methods based on those principles do not require expensive equipment, making them suitable for workshop alignment. One of the drawbacks of this method may be the accuracy equal to that of an azimuth measurement in an environment usually filled with clutter and reflective surfaces that may distort the measurement. The improvement in accuracy can be achieved by increasing the number of radars that observe alignment targets and, when possible, setting up multiple calibration targets within the common field of view of multiple radars and then finding the optimal solution for all misalignment angles in the ECU [66]. The increased accuracy of this solution comes at the cost of a massive increase in space requirements, and it may only be suitable for some assembly lines and even a workshop. It can be performed only on a system with many radars, overlapping fields of view and targets at the field of view’s intersections.

Increasing the number of targets allows us to use algorithms that may not require an azimuth measurement. It will only estimate the radar position and orientation based on a range measurement, as described in the first part of [67]. Using a range measurement of three radar targets to calculate misalignment may result in higher estimation accuracy, depending on the range resolution of the radar and the placement of the targets. Multiple targets or repeated measurements of radar targets at different positions may be used to estimate both azimuth and elevation misalignment [68,69,70]. The increasing number of targets may result in better accuracy if the angular error distribution of radar angle measurement is a zero mean, which cannot be guaranteed since multipath reflections may disrupt the angle estimation, especially for elevation. As with any method, multiple targets may not be feasible due to workspace limitations.

The mentioned methods, when used with passive targets such as spherical or corner reflectors, are prone to inaccuracies caused by stationary objects and surfaces in the vicinity that can generate reflection, which will disrupt the returning phase of a signal. To avoid problems caused by this effect, using active targets such as the transponder described in [71] may be beneficial. It can generate a delayed reflection with a shifted frequency, simulating the target at a range far above that of other equipment and a velocity that is unique in the surrounding environment. A device like this can improve static alignment performance by generating targets for range-Doppler bins unoccupied by other objects. Such transceivers increase the cost of a calibration station.

Angular accuracy in challenging conditions may not be sufficient to estimate high-quality misalignment angles. The symmetrical gain pattern of most automotive antennas can give insight into alignment angles based on the amplitude of the radar wave transmitted to the target—which should be the highest when the target is strictly at the centre of the field of view. Methods for estimating misalignment based on signal power at target locations are described in [72,73,74]. With carefully designed targets, there is no need to use external measurement devices, as radar should be capable of determining the received power. The method in [75] describes alignment based on two targets that generate the same amplitude placed symmetrically above and below the radar. In cases where the radar is not misaligned in elevation, the amplitude from both targets should be almost identical. The radar will observe a more substantial reflection from the target below the radar if the sensor is misaligned towards the ground. Using marks that provide a sharp angular response may even further improve the visibility of amplitude patterns. One such target is the flat plate mentioned by [64]. In [76], calibration with a flat plate is further improved by compensating for azimuth-angle inaccuracies caused by sidelobes of the reflection spectrum. Alignment methods based on the power of the transmitted or received signal may come at an increased cost of equipment, but they have significant advantages due to the fact that they may be used to align the radar even if it cannot estimate the angle itself.

The alignment problem can be treated purely as a mechanical problem described in [77]. In that case, it may be assumed that it is sufficient to measure the position of the radar with precise measurement equipment and either physically correct the position or compensate for the misalignment angle in the sensor software. As Abou-Jaoude points out in [64], that method requires the disassembly of any part that may cover the radar, and it will not be able to detect or compensate for misalignment caused by the fascia or squint of the antenna.

### 4.2. Dynamic Alignment

Dynamic alignment requires the continuous unsupervised operation of the algorithm, and to satisfy functional safety standards, it should be used to maintain high accuracy for radar detections in the lifetime of the sensor [12]. The further part of this subsection shows an overview of dynamic alignment algorithms categorised by the requirement for an additional accelerometer sensor and independence from measurement devices other than radar.

#### 4.2.1. Accelerometer-Based Dynamic Alignments

Preston and Olmstead submitted a patent application for alignment methods based on 3D accelerometers mounted on radars and a vehicle frame to determine the misalignment angle based on the difference between acceleration vectors [78]. More straightforward implementations of alignment with inertial sensors are described in [79] for generic front sensors and in [80] for a front radar. In [81], Steinbuch and Schnabel describe an invention capable of changing the direction of an antenna’s emission to compensate for misalignment. The relatively low cost, size and accuracy of modern accelerometers could—with additional signal filtration—provide high accuracy in alignment estimation. The drawback of these accelerometer-based or IMU-based methods is that they require the precise mounting of accelerometers on radars and calibration performed on each sensor to ensure that they provide correct measurements relative to the radar body. In the case of radar-accelerometer mounting, it could be performed as part of radar manufacturing. The acceleration sensor mounted on the vehicle frame would still need to be calibrated during vehicle assembly, which could increase the cost of production.

#### 4.2.2. Radar Measurement-Based Dynamic Alignments

While the methods described in other subsections use external sensors, equipment and road information, it is possible to estimate the misalignment angle of a sensor based solely on radar measurements. Depending on the radar design, some measurements may provide more accurate data than others. Those differences between radar types require a careful selection of the methods used for alignment.

In cases where the radar cannot determine the elevation angle of objects or the accuracy of the elevation angle is subpar, the method described in [82] can be used. It is based on a delay in the received wave amplitude from the ground reflection. In cases where the radar is misaligned towards the ground, the delay in the returning wave’s peak is shorter. Based on the difference in the delay observed in an adequately aligned sensor, it is possible to estimate the direction of misalignment. Although this method does not require radar detections to work, it is necessary to consider that the precise measurement of the delay may not be available in every radar design.

Another group of algorithms uses range and azimuth measurements to calculate misalignment based on trigonometric dependencies. In [83], Ameen and Ryan use detections of other moving vehicles observed in two instances of time to estimate misalignment. Kim’s method, described in [84], uses a target in a common field of view with two radars and only requires range measurements. Another approach, presented in [85], is to observe a stationary track and use its range and azimuth measurements collected at different times. In the case of this method, it is crucial to ensure that the measurements used are taken when the ego vehicle travelled a significant distance. Otherwise, if the measurements are taken close to each other, it may be difficult to find unique solutions to the equations. Algorithms based on two radar measurements require the observation of a target in the common field of view. The overlapping view may not be wide enough to provide such observations, especially considering that both sensors should be able to pinpoint an exact point on the target reported by the other radar. One of the reasons for the inaccuracies of algorithms in this group is that everyday objects in the surroundings have complex geometry, and depending on the perspective of the radar, they reflect radar waves in different locations. This causes the common target not to be in the exact location, contributing to the estimation error.

The output of the tracking algorithms can be used instead of using direct radar measurements that may not be accurate. In [86], the trajectory of a tracked object is compared to the trajectory of an ego vehicle. The differences between the two can be used to estimate the misalignment angle of a sensor. Another approach is used in [87], where the algorithm uses the tracking states of two radars observing the same object to determine the angles of misalignment.

Misalignment angles can be calculated based on the difference between the known ego-vehicle velocity vector and the perceived motion estimated based on stationary targets surrounding the radar. Such methods are presented in [88,89], where azimuth and range-rate measurements from multiple detections are used to estimate the sensor velocity vector. In [90], additional information from the gyroscope is used to jointly align the radar and vehicle odometry. In [91], the target velocity vector is calculated for objects in the common FoV of two radars, and the difference between two observed velocity vectors is used to estimate misalignment.

The next group of alignment estimation methods uses Doppler velocity with spatial measurements. The method for estimating the calibration matrix that eliminates spatial inaccuracies of detections is described in [92]. The finding of alignment angles can be solved by minimising errors in the range-rate equation, Equation (12), with [93] or [94]. High-precision sensors with azimuth and elevation measurements enable the use of the method described in [95] to estimate azimuth and elevation misalignment with a correction factor for the measured vehicle velocity. This method is extended with the roll angle misalignment estimation in [96]. If given enough stationary detections, algorithms from this group may provide an accurate and instantaneous estimate. Depending on the selected method, the algorithm may be computationally expensive and unsuitable for computation using embedded processors. Nonlinear optimisation may be sensitive to data inaccuracies. Problems may arise when the algorithm, accidentally, is given slow-moving objects or detections with a low angular accuracy instead of stationary detections.

### 4.3. Track Alignment

Track-alignment algorithms strongly resemble dynamic-alignment algorithms, as both must be executed on the radar or vehicle ECU. Many dynamic-alignment algorithms could be modified to consider predefined environmental patterns in order to provide a higher estimation accuracy in a shorter time. For example, it could be easy to recognise shapes made of corner reflectors or barriers/targets that are guaranteed to form straight lines along the vehicle trajectory [97]. The method described in [98] is based on the geometry of the surrounding barriers that need to form a line parallel to the road surface in order to determine the vertical misalignment of the sensor. Although the authors claim that the method is unsupervised, the input data selection criteria are not described in detail. This leads to the conclusion that, for example, inclined barriers may pose a problem for the algorithm, making it only suitable for specific roads. With a proper road target setup, the accuracy and time to provide a high-precision estimation of misalignment angles could outperform dynamic alignment. However, the required space for calibration setup may be infeasible in most use cases.

### 4.4. Post-Factum Alignment

Some alignment methods require complex computations that are too computationally expensive to be performed on an embedded processor or require the storage of radar measurements for a considerable period. Although they are not fit to be part of radar devices or alignment equipment, they may provide more accurate alignment angles that could be used as reference values for data analysis and development.

An example of such an algorithm is one based on collecting scenarios of driving around stationary targets that can, after data collection, be transformed into a grid map in a global coordinate system. Grid maps may be recalculated, assuming various misalignment angles. For each misalignment angle, a cost function is calculated to score the blurriness of stationary objects in the resulting grid. The actual misalignment angle is used for compensation when the stationary objects on the grid map are the sharpest [67]. In another method, Suzuki et al. use Markov-chain Monte Carlo to estimate the misalignment angle as a correction table for detections across different azimuth angles [99]. Methods used to match trajectories of objects observed via multiple radars, which were not originally designed for automotive use, could be used to estimate misalignment in a system where radars have overlapping fields of view [100,101,102]. As ADAS systems become more complex and require various sensors, aligning radar data with other measurements becomes crucial. Alignment and data fusion from radar, lidar and a stereovision camera are presented in [103].

## 5. Algorithm Evaluation

This section outlines the fundamental approach to assessing radar alignment algorithms. Section 5.1 details the datasets used for the assessment. The algorithm proposed as a baseline for radar alignment performance in this evaluation is addressed in Section 5.2, and its evaluation results are shown in Section 5.3.

### 5.1. Dataset

A proper assessment of alignment-algorithm performance should use an open dataset, facilitating the comparison of results from various algorithms developed by different researchers. For static and track-alignment algorithms, using a common dataset might be unfeasible due to specific environmental, equipment and calibration needs. In contrast, radar detection-based dynamic alignment algorithms should operate under standard driving conditions, making radar data from test drives adequate for their evaluation. This also applies to certain post-factum alignment methods that do not require particular test setup requirements. Currently, most radar manufacturers do not publicly share extensive radar specifications and data. However, a slight trend towards sharing with the scientific community has been observed with the introduction of new open sensor datasets.

One such example is the RadarScenes dataset [104], which contains longer sequences of radar measurements from real-world drives and is available under the Creative Commons Attribution Non-Commercial Share Alike 4.0 International Licence. This dataset contains vehicle kinematics measurements and radar detections from four radar sensors mounted on the same vehicle. The sensors are mounted with a focus on the front of the vehicle at declared boresight angles of −85, −25, 25 and 85 degrees. The horizontal FoV of the sensors is about 120 degrees, measurements are collected approximately every 60 ms and this sensor has no elevation measurement capability. Researchers may be interested in the fact that image sequences and detection labels are also available for this dataset, which can make the use of this dataset easier.

It should be emphasised that, for algorithms aiming to exclude non-stationary detections, the ”Other” class can be contaminated with slowly moving objects. Consequently, additional algorithmic filtering may be required to utilise sensitive algorithms designed for stationary environments. In the following subsections, the presented data set is used to deliver KPIs for the chosen alignment method.

### 5.2. Baseline Algorithm

The reference algorithm should be robust to ensure precise estimations while also being easy to implement and effective in most scenarios. To reduce the number of parameters to consider during implementation, a straightforward post-factum algorithm was chosen. This algorithm aims to solve the range rate model described in Equation (12) for a stationary environment ($T_{x}=0$ and $T_{y}=0$) in order to estimate two parameters: the speed compensation factor ($k_{V_{x}}$) and azimuth misalignment. The equation used to estimate these parameters is as follows:

(32) $$f\left(k_{V_{x}},\alpha_{m}\right)={\dot{r}}_{n}-\left(V_{\omega_{x}}-k_{V_{x}}V_{x}\right)\cos\alpha_{n_{\mathrm{VCS}}}+V_{y}\sin\alpha_{n_{\mathrm{VCS}}}=0,$$

where $V_{\omega_{x}}$ is a component related directly to the vehicle yaw rate (as well as $V_{y}$, which could also be denoted as $V_{\omega_{y}}$).

#### 5.2.1. Selection of Stationary Detections

The steps in this subsection aim to identify high-confidence stationary detections independently for each sensor. To preliminarily select detections of interest, those labelled the “other” class and recorded when the vehicle speed exceeded 5 m/s with an absolute acceleration below 0.5 m/s^2^ were chosen. The subsequent step involved a voting transformation on a grid, factoring in the azimuth compensated with the sensor heading and the normalised range rate domain (normalised according to the vehicle’s absolute speed). The constructed grid has a width of 100 azimuth bins and a height of 200 range-rate bins. Each detection increments counter in the bin, which is related to the detection’s azimuth and range-rate measurements. From each column of the azimuth bin, the range-rate bin with the highest counter is picked. Only detections contributing to the selected bin counter proceed to the next step. The actions performed aim to create a high-accuracy estimation of the stationary detection curve in the azimuth–range rate domain, which is ensured due to the fact that most of the radar detections are constructed on stationary objects, especially when all the moving classes labelled in the dataset were removed during the preselection phase.

**Figure 15:**
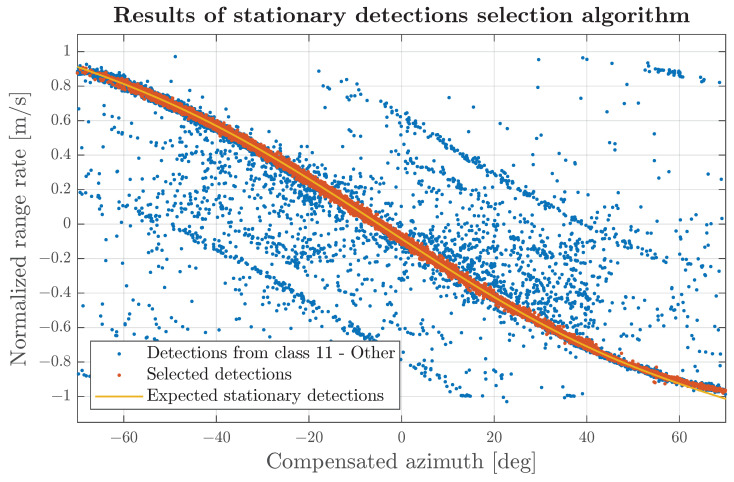
Illustration depicting the initial inputs for the stationary detection selection algorithm (blue), the chosen detections (orange) and the expected stationary detection curve (gold). Radar detections from RadarScenes—Sequence 1.

Detections selected in the previous phase were used to fit a fifth-order polynomial. Fitted polynomial functions were applied to estimate the expected range rate of stationary detections. The range rate for detections previously categorised as “other” was evaluated against the projected polynomial curve, and only detections with an absolute deviation of less than 0.05 m/s were considered for subsequent calculations. The working principle of the algorithm is presented in Figure 15.

#### 5.2.2. Solving Non-Linear Equation with Nonlinear Least Squares

The parameters of Equation (32) were estimated using the well-established nonlinear least-squares (NLS) technique. It is essential to derive the Jacobian matrix of the given model, built from the derivatives of Equation (32):

(33) $$\frac{𝜕f}{𝜕k_{V_{x}}}=V_{x}\cos\left(\alpha_{b}+\alpha_{n}+\alpha_{m}\right),$$

(34) $$\frac{𝜕f}{𝜕\alpha_{m}}=\left(V_{\omega_{x}}-k_{V_{x}}V_{x}\right)\sin\left(\alpha_{b}+\alpha_{n}+\alpha_{m}\right)+V_{y}\cos\left(\alpha_{b}+\alpha_{n}+\alpha_{m}\right).$$

The Jacobian matrices are constructed utilising the partial derivatives from Equations (33) and (34), based on 256 randomly chosen stationary detections for each sensor, thus forming a 256 × 2 matrix for a single sensor.

**Figure 16:**
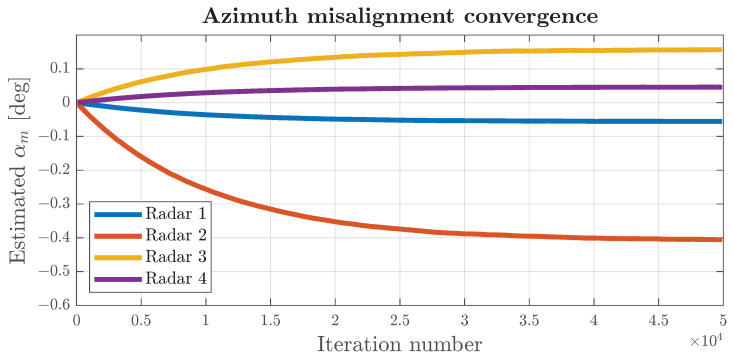
Illustration depicting azimuth misalignment convergence for each sensor, performed on radar detections from RadarScenes—Sequence 1.

The algorithm operates iteratively by choosing a random subset of detections from each radar and refining the estimates. To precisely compute the speed-compensation factor, which is a parameter shared among all vehicle sensors, the algorithm alternates between different sensors in each iteration. To achieve a reliable estimation in the parameter-refinement phase, the algorithm uses the values $U,\Sigma ,V^{\prime }$ obtained from the singular value decomposition (SVD) of the Jacobian. During each algorithm iteration, the corresponding sensor values are updated as follows:

(35) $$\Delta_{b}=V\Sigma^{-1}\left(U^{\prime }\eta \Delta_{L}\right),$$

where $\Delta_{b}$ represents the update step of the estimated parameters, and η is a parameter that indicates the desired fraction of descent from the actual residual, denoted as $\Delta_{L}$. The parameter η can be interpreted similarly to a learning rate in machine learning, and in this instance, it was chosen to be $10^{-4}$ in order to ensure a gradual yet consistent convergence towards the final values of the estimated parameters. The resulting convergence is depicted in Figure 16.

### 5.3. Key Performance Indicators and Results

**Figure 17:**
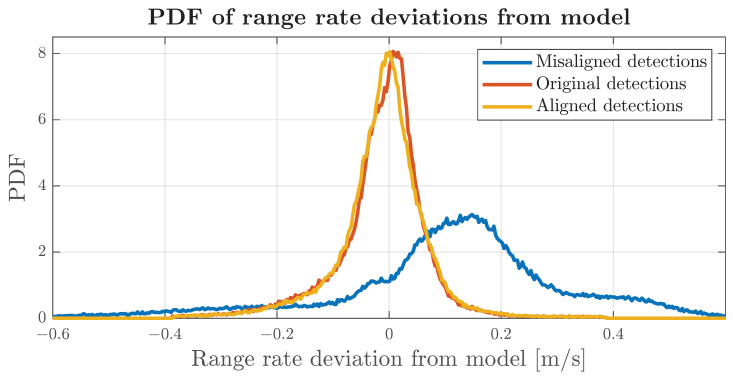
Probability density function of the range-rate deviations from the model accumulated across all vehicle radars. Sensors deliberately misaligned by following $\alpha_{m}$: 3, −3, −2, −1 degrees (blue), original mounting angles (orange) and correctly aligned sensors (gold). RadarScenes—Sequence 1.

One of the difficulties in developing KPIs for radar alignment lies in ensuring that they can be computed with all types of automotive radars. A particularly valuable KPI would be the root mean square error (RMSE) of the GCS position error in narrow objects. These objects can offer insight into how the identified alignment angle improves the GCS position of stationary targets. To effectively exploit such a KPI, researchers would require radars that could report large quantities of detections, offer high azimuth resolution and provide outputs at a relatively high frequency. However, with the RadarScenes dataset, the number of matched objects between scans was too small to draw meaningful conclusions. The most straightforward and transferable KPIs seem to be associated with the range-rate error. Even low-grade automotive sensors seem to provide quite reliable range-rate estimation to accurately measure the velocities of surrounding targets. Figure 17 shows that, if the sensors are misaligned, the error distribution of the range-rate equation will greatly vary. This knowledge can be used for the implementation of KPIs that can be transferred to different sensor models.

The proposed reference algorithm was applied to Sequence 1 of the RadarScenes dataset, resulting in the values shown in Table 2. These values were used to align all detections within Sequence 1 and compute the root mean square error (RMSE) based on the discrepancies between the range-rate model estimates and the actual range rate measurements. The errors produced were filtered to exclude samples beyond 4σ, and then the filtered samples were used to determine the RMSE, skewness and kurtosis. The same procedure was performed for the detections that had sensors intentionally misaligned by $\alpha_{m}$: 3, −3, −2, −1 degrees and the original detections. The results of this comparison are presented in Table 3.

The procedure used in this subsection can be reproduced for different algorithms and possibly different datasets to show the difference in the range-rate errors of stationary detections. The algorithm presented here is simple enough to be reproduced and used as a comparator even with a dataset that was not considered in this publication.

## 6. Summary

The number of features that can help drivers travel safely is growing, and their complexity is increasing. In the course of this evolution, even the most sophisticated algorithms will reach their reliability limits if they are not provided with accurate observations of the environment. An advancement in sensor capabilities, driven by those requirements, will start to result in devices with a measurement accuracy higher than that of their mounting on a vehicle. To ensure that safety systems operate with maximum reliability, it is vital to minimise sensor misalignment on the assembly line and monitor it regularly. Unexpected conditions during the lengthy lifetime of a sensor in a car make self-calibration and self-diagnostic sensor capabilities that maintain high-quality measurements and ensure the fail-safe operation of the system crucial.

The most noticeable impact of angular-mounting inaccuracies is a systematic and constant error that impacts all detections provided via a radar. In addition to position inaccuracies, leading to decreased state-estimation accuracy, these can contribute to the incorrect classification of the target’s road lane. The physical offset of a sensor may also increase blind zones around a vehicle, create partial or complete sensor blockage, make distinguishing relevant targets from the clutter impossible or significantly reduce the maximum range of detection. Ensuring that those problems are minimised is beneficial, especially in the case of radar visibility for pedestrians that have a relatively low RCS compared to the surrounding clutter. Angular errors also affect heading estimation and fusion algorithms, causing an exponential increase in track splitting, which may cause the system to be non-operational.

Multiple countermeasures can be implemented to ensure the proper adjustment of a radar in a vehicle. Sensors can be mounted on specialised brackets that automatically or manually compensate for inaccuracies in mounting. The adjustment angle can be estimated in the calibration procedure in the factory or vehicle workshop and during a drive on a specially designed track or in an unsupervised manner on public roads.

The emergence of new open data sets provides interesting opportunities to compare the performance of different algorithms. The alignment algorithm presented in this publication, together with the KPIs that were implemented to showcase alignment performance, can be used as a baseline to compare other methods.

The impact of radar misalignment can be design-specific, and due to multiple sensor and system compositions, it is impossible to select the best method and provide exact values at which the system becomes unreliable. Compared to other fields in automotive research, there is a limited amount of available literature that focuses on the alignment of radar sensors. However, the emergence of open radar datasets and the increase in computational power available in vehicle ECUs may provide interesting research opportunities.

## Figures and Tables

**Table 1:** Parameter values for Land Clutter Model [19,22].

Terrain Type	*A*				*B*	*C*	*D*
Frequency [GHz]	15	9.5	5	3	All	All	All
Soil, sand, and rocks	0.05	0.025	0.0096	0.0045	0.83	0.0013	2.3
Grass and crops	0.079	0.039	0.015	0.0071	1.5	0.012	0.0
Trees	0.019	0.003	0.0012	0.00054	0.64	0.002	0.0
Urban	2.0	2.0	0.779	0.362	1.8	0.015	0.0

**Table 2:** Outputs of the proposed reference algorithm for RadarScenes—Sequence 1.

Radar	$\alpha_{m}$ [deg]	$k_{V_{x}}$ [1/1]
1	−0.0563	1.001430
2	−0.4072	
3	0.1563	
4	0.0462	

**Table 3:** KPI results of the proposed reference algorithm for three considered cases: misaligned detections, original detections and aligned detections. The deviations from all radars were jointly evaluated according to the KPIs. Based on RadarScenes—Sequence 1.

	Misaligned Detections	Original Detections	Aligned Detections
RMSE	0.2398	0.0841	0.0831
Skewness	−0.6984	−0.5062	−0.3702
Kurtosis	4.6539	6.6103	6.6366

## Data Availability

The simulations in Section 2 were implemented with Monte Carlo methods as described in the article. If needed, additional details will be provided upon contact with the author. The algorithm evaluation in Section 5 was performed using the RadarScenes dataset, which can be downloaded from https://doi.org/10.5281/zenodo.4559821 (for data description and licence details, see https://radar-scenes.com).
